# Novel Monolithic CAVET-HEMT Integration for Inverting-Switch Operation

**DOI:** 10.1021/acsomega.6c01803

**Published:** 2026-04-24

**Authors:** Weng-Hooi Tan, Haocheng Zhao, Muhammad Farizuan, Amirul Firdaus, Hiroshi Kawarada, Tao Tao, Shaili Falina, Mohd Syamsul

**Affiliations:** † Institute of Nano Optoelectronics Research and Technology (INOR), 26689 Universiti Sains Malaysia, Sains@USM, Bayan Lepas 11900, Pulau Pinang, Malaysia; ‡ Collaborative Microelectronic Design Excellence Centre (CEDEC), Universiti Sains Malaysia, Sains@USM, Bayan Lepas 11900, Pulau Pinang, Malaysia; § Faculty of Science and Engineering, Waseda University, Tokyo 169-8555, Japan; ∥ Power Diamond Systems Inc., Shinjuku, Tokyo 169-0051, Japan; ⊥ School of Electronic Science and Engineering, 12581 Nanjing University, Nanjing 210093, China

## Abstract

This work presents a monolithic GaN-based CAVET-HEMT, a novel device that integrates a channel aperture vertical electron transistor (CAVET) with a lateral high electron mobility transistor (HEMT) into a single compact structure. The internal cascade allows the CAVET to deliver a voltage-modulated signal to the HEMT channel with 180° phase inversion, producing the device’s distinctive inverting-switch behavior, in contrast to the normal switching operation of conventional HEMTs. This internal integration eliminates the need for external interconnections, reducing parasitic losses while enabling enhanced voltage control and faster device response. Electrical characterization highlights the superior performance of the CAVET-HEMT. It achieves a saturation current (*I*_D,sat_) of 0.707 A/mm, significantly higher than the 0.290 A/mm of the HEMT, and exhibits a peak transconductance (|*g*_m_|) of 4.296 S/mm, compared to 0.064 S/mm for the HEMT. The triode operating range (Δ*V*_triode_) is compressed to 1.15 V, reflecting a 5.43× improvement in gate-voltage sensitivity relative to HEMTs. Additionally, the device demonstrates a lower on-resistance (*R*_on_) of 11.61 Ω·mm, while the output conductance (*g*_d_) reaches 0.088 S/mm, indicating strong current-driving capability. Despite increased parasitic capacitance, high-frequency operation is maintained with *f*_T_ = 3.5 GHz and *f*_max_ = 6.5 GHz, slightly exceeding HEMT performance (by ∼13% and ∼8.3%, respectively) and confirming that the CAVET-HEMT effectively enhances transconductance without being limited by added capacitances. Overall, the proposed CAVET-HEMT conceptually combines superior current-handling, sharp transconductance, high-frequency performance, and inverting-switch functionality within a monolithic architecture. By embedding cascading behavior directly into the device structure, it opens new possibilities for compact, high-speed, and high-power switching and even logic applications (acting as a NOT logic gate). This approach represents a paradigm shift in GaN device design, demonstrating that integrated vertical-lateral architectures can achieve performance levels traditionally requiring multiple discrete stages, while offering new functionality and operational versatility.

## Graphical abstract

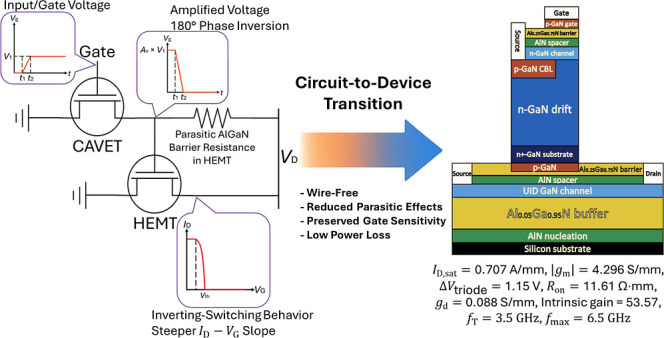

## 1 Introduction

High electron mobility transistors (HEMTs) have been widely studied as a key platform for achieving fast switching, high current density, and low on-resistance. HEMTs are heterostructure-based devices, originally developed on AlGaAs/GaAs material systems and later extended to wide-bandgap semiconductors such as AlGaN/GaN.^1–4^ The HEMT revolutionized high-frequency and high-power electronics by utilizing a two-dimensional electron gas (2DEG) formed at the heterointerface, which enables exceptionally high carrier mobility, rapid switching, and low on-resistance. Over time, HEMT architectures have undergone continuous refinement through advancements such as p-GaN gate technology,^5^ cap layer engineering,^6^ and optimized back-barrier designs,^7^ each aimed at improving breakdown voltage,^8^ transconductance, and overall device efficiency. In amplifier applications, these structural and material advantages are theoretically expected to yield faster frequency response, higher gain, and enhanced efficiency, particularly in RF^9^ and microwave systems.^10^ Consequently, the adoption of HEMTs could significantly expand the amplifier performance matrix in terms of speed, frequency capability, and signal fidelity.

Parallel to these advancements, the current aperture vertical electron transistor (CAVET) emerged as a promising architecture to address the current-handling limitations of conventional lateral HEMTs. Early CAVET designs introduced a vertical conduction pathway through the GaN layer, enabling electrons to flow from source to drain in the vertical direction while maintaining a high-mobility lateral channel region beneath the gate. This configuration effectively combined the superior material properties of GaN with the benefits of vertical current transport, thereby achieving higher breakdown voltage, improved thermal dissipation, and enhanced current density compared to purely lateral HEMT devices.^11^ Subsequent advancements in CAVET technology have further refined this concept through a series of structural innovations, including dielectric isolation techniques,^12^ trench-based channel architectures,^13^ and engineered doping strategies such as stepped^14^ or graded n-type pillars within the drift region^15^ to confine current flow precisely within the active channel. These refinements collectively improved electrostatic control, minimized leakage current, and enhanced device robustness under high electric-field operation. In exchange for their high-performance capabilities, CAVETs face significant material processing and fabrication challenges, particularly in achieving reliable regrowth interfaces and precise vertical alignment.^11^

The need for improved electrostatic control, voltage amplification, and gain modulation has inspired exploration of internal cascading mechanisms within single devices.^16,17^ Cascaded architectures, where one functional stage drives another internally, can achieve intrinsic voltage amplification and stronger channel modulation without the complexity, parasitic losses, and power penalties associated with external multistage circuits.^18^ This concept offers a pathway to combine high current-handling, strong electrostatic control, and compact device integration, potentially enabling novel switching behaviors that cannot be realized in conventional devices.^19^

Despite extensive research on HEMTs and CAVETs individually, no prior work has realized their monolithic integration. The proposed CAVET-HEMT represents a completely novel device architecture, embedding a vertical CAVET directly onto a lateral HEMT. This integration is not a mere combination of the two devices’ capabilities; rather, it creates new operational dynamics by internally cascading the CAVET and HEMT within a single structure. Through this internal cascade, the CAVET region functions as a voltage-amplifying component, directly controlling the gate of the underlying HEMT channel. This monolithic arrangement eliminates external interconnections, reduces parasitic losses, and enables a unique inverting-switch behavior that is fundamentally different from conventional HEMTs. Unlike standard HEMTs, where the device turns on when the gate voltage exceeds the threshold, the CAVET-HEMT conducts at gate voltages below its threshold and turns off when the gate exceeds it. This inverted switching functionality demonstrates how monolithic cascading can fundamentally alter the operational characteristics of the device.

The objective of this research is to present the design and operating principle of the CAVET-HEMT, highlighting how the integration of vertical CAVET and lateral HEMT enables internal voltage amplification, enhanced electrostatic control, and inverting-switch operation in a single device. By consolidating the functions of multiple transistor stages into one monolithic structure, the CAVET-HEMT offers a pathway to higher current-handling, stronger gate control, and compact device footprints, representing a new paradigm in GaN transistor design that is unattainable with standalone HEMTs or CAVETs.

## 2 Validation

We validated our simulations against a p-GaN-gated AlGaN/AlN/GaN HEMT on a silicon substrate reported in literature.^20^ Figure 1 illustrates the validation of the simulation with respect to experimental results. This validation step allowed us to calibrate the Silvaco-Atlas^21^ configuration to ensure that the simulated results closely match the measured device characteristics. Based on this calibration, we established the physical model set required for accurate TCAD representation. Shockley–Read–Hall (SRH) and Auger recombination mechanisms were included to capture temperature-dependent carrier recombination through defect states under both on-state and off-state bias conditions. Carrier mobility was modeled using the parallel-electric-field dependent (FLDMOB) model for high-field behavior, complemented by the Albrecht and GaNsat models to account for concentration-dependent low-field mobility specific to GaN. Additionally, a polarization model must be incorporated to represent spontaneous and piezoelectric polarization effects. In theory, spontaneous and piezoelectric polarization in AlGaN/GaN structures generate high interface charge; however, in real devices, this charge is effectively reduced due to nonideal effects such as interface trap states, defect-related charge compensation, surface states, and partial strain relaxation in the epitaxial layers. Hence, TCAD simulations typically incorporate a scaling factor to better reflect the physically realizable polarization charge density. The scaling factor of 0.68 was determined through a calibration process during the validation phase, where the polarization scaling factor was systematically adjusted to achieve the best agreement between simulated and reference electrical characteristics, particularly in terms of threshold voltage, drain current, and transconductance. This calibrated value ensures that the simulation accurately captures the electrostatic behavior of the device while maintaining consistency with experimentally observed performance trends. Collectively, these models provide a physically consistent and robust framework for simulating carrier transport and polarization phenomena in GaN-based HEMTs.

**Figure 1:**
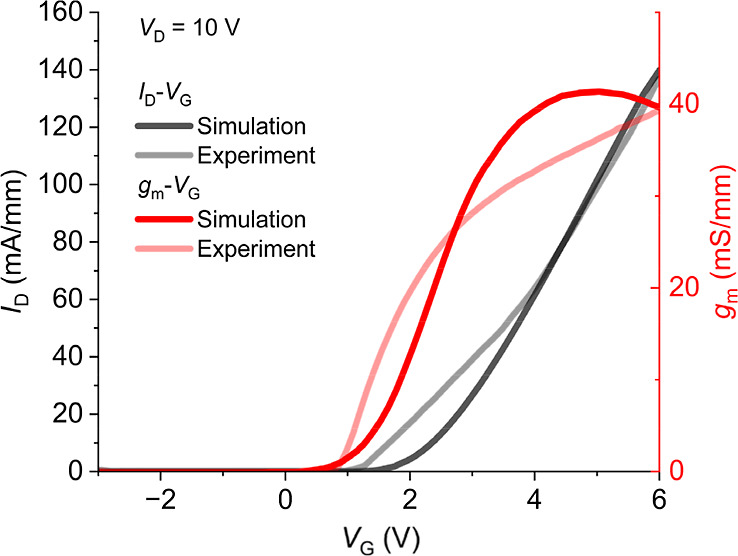
Experimental vs simulated output behavior of the GaN-based HEMT upon calibration of simulation models.

## 3 CAVET-HEMT

This work proposes a monolithic GaN-based CAVET-HEMT device that integrates a CAVET onto a HEMT within a single structure, as illustrated in the three-dimensional schematic in Figure 2. Table 1 summarizes the key parameter settings of the proposed CAVET-HEMT device, providing a detailed overview of its structural and operational specifications. To highlight the operating principle, Figure 3 presents a direct comparison between the proposed CAVET-HEMT and a HEMT at both the circuit and device levels.

**Figure 2:**
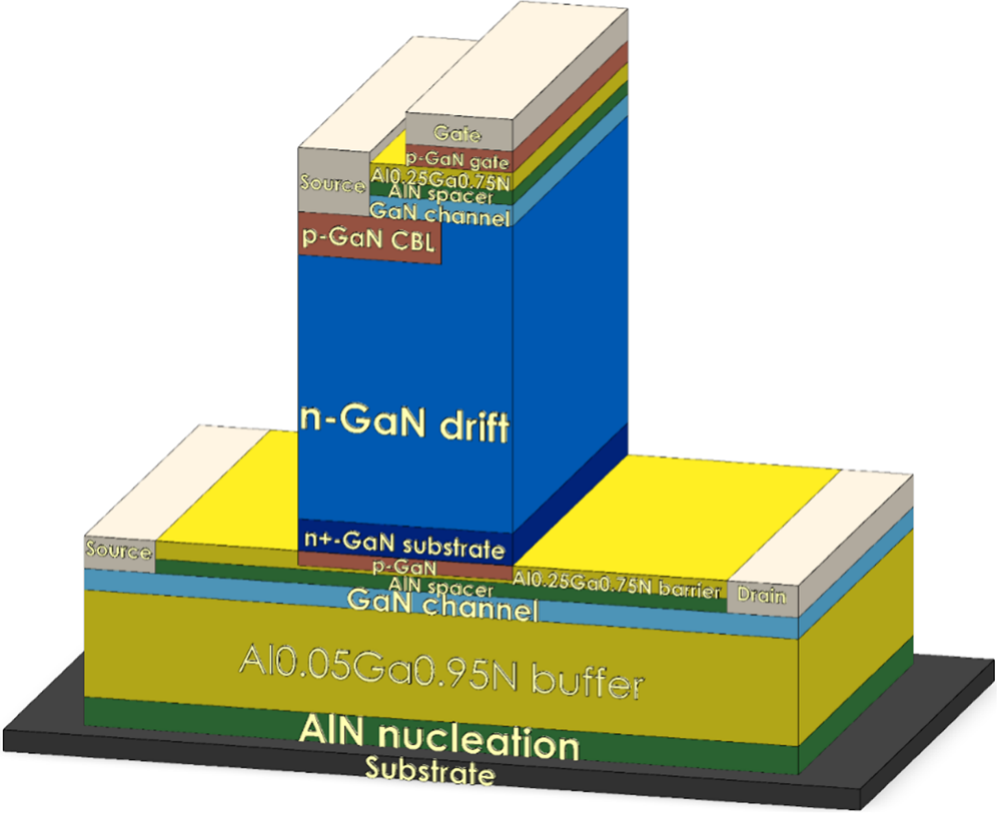
Schematic illustration of the three-dimensional CAVET-HEMT structure.

**Table 1:** Design Parameters of the Proposed CAVET-HEMT Device

device part	parameter	vertical dimension (nm)	horizontal dimension (μm)	doping type/concentration (cm^–3^)
electrode	gate-to-drain spacing	-	3	-
	source (HEMT) to source (CAVET) spacing	-	2	-
	gate-to-source (CAVET) spacing	-	0.5	-
	drain length	-	1	-
	source (HEMT) length	-	1	-
	source (CAVET) length	-	1	-
	gate length	-	1.5	-
CAVET	p-GaN gate	39	1.5	p-type/1 × 10^18^
	Al_0.25_Ga_0.75_N barrier	10	2	UID
	AlN spacer	1	2	UID
	GaN channel	100	2	UID
	p-GaN CBL	500	2	p-type/1 × 10^18^
	n-GaN drift layer	3500	3	n-type/1 × 10^17^
	n^+^-GaN substrate	500	3	n-type/5 × 10^18^
HEMT	p-GaN	20	3	p-type/2.5 × 10^18^
	Al_0.25_Ga_0.75_N barrier	25	8	UID
	AlN spacer	1	8	UID
	GaN channel	100	10	UID
	Al_0.05_Ga_0.95_N buffer	1000	10	UID
	AlN nucleation	274	10	UID

**Figure 3:**
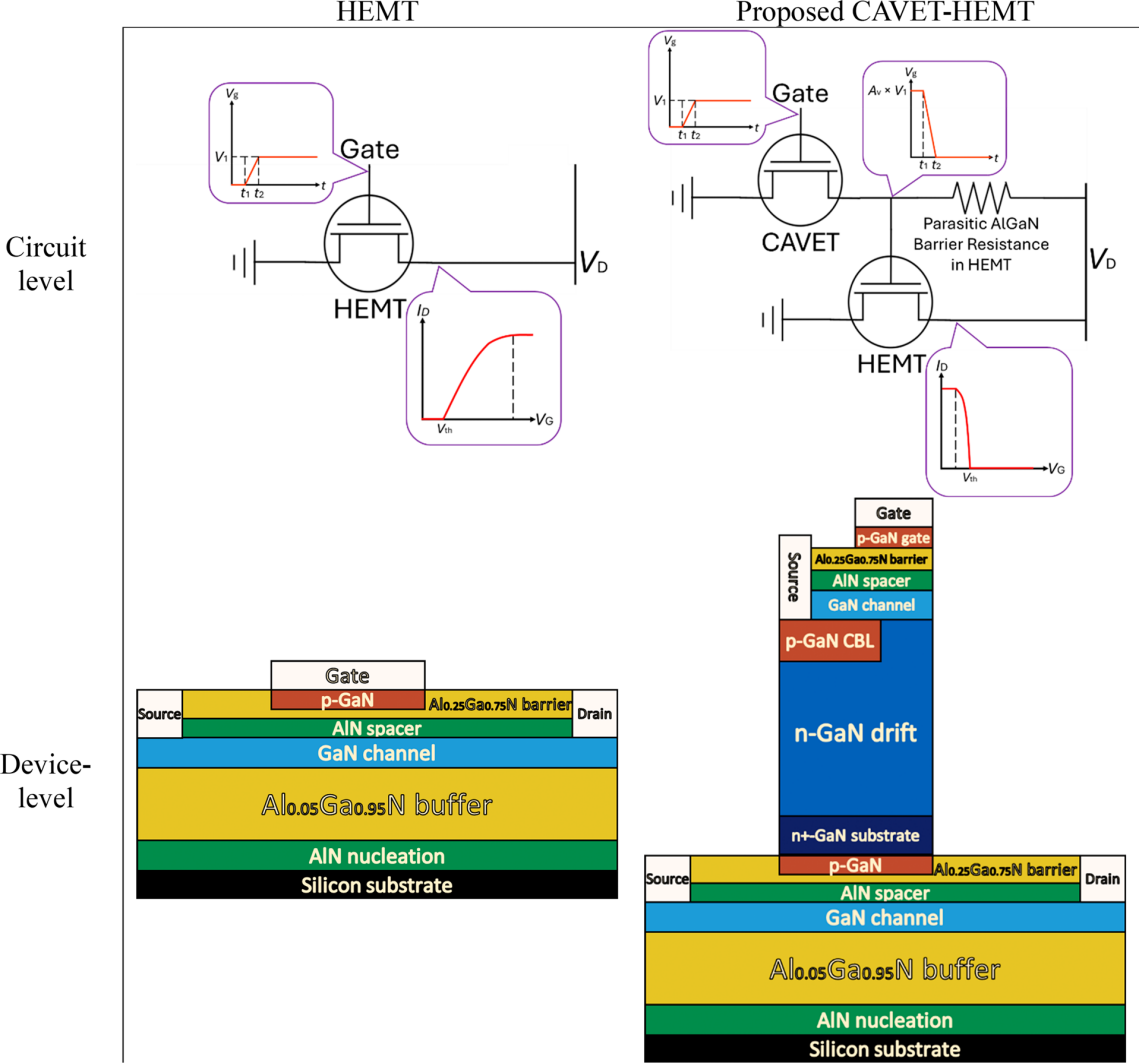
Circuit- and device-level comparison between CAVET-HEMT and HEMT.

At the circuit level, cascading the CAVET onto the HEMT forms the complete CAVET-HEMT configuration. The CAVET is deliberately engineered as a voltage-amplifying stage with a deliberate 180° phase inversion. Small variations in the CAVET gate voltage are translated into amplified voltage changes at the effective p-GaN region of the lateral HEMT, such that a modest increase in the CAVET gate voltage produces a substantially larger reduction in the HEMT gate potential. In operation, increasing the input gate voltage beyond the threshold (*V*_th_) fully depletes the HEMT channel and drives the device toward the OFF state. This behavior contrasts with a HEMT, where gate bias above *V*_th_ enhances channel conduction and strengthens the ON state. Together, these mechanisms compress the *I*_D_–*V*_G_ operating range and enable the CAVET-HEMT to function as an inverting switch. From a logic perspective, the device exhibits NOT-gate behavior, in which a high input produces a low output.

At the device level, the monolithic CAVET-HEMT eliminates external wiring and interstage connections. This integration reduces parasitic effects and lowers gate-drive requirements. By leveraging the intrinsic advantages of GaN, the CAVET-HEMT enables efficient high-speed switching while maintaining a compact footprint and enhanced reliability, making the architecture inherently suitable for high-frequency operation.

To elucidate the operating mechanism of the CAVET-HEMT device, the conduction band profiles under three bias conditions are presented in Figure 4a–c. The device operation is fundamentally governed by the depletion or formation of the two-dimensional electron gas (2DEG) at the AlN/GaN interfaces in the lateral HEMT and vertical CAVET regions.

**Figure 4:**
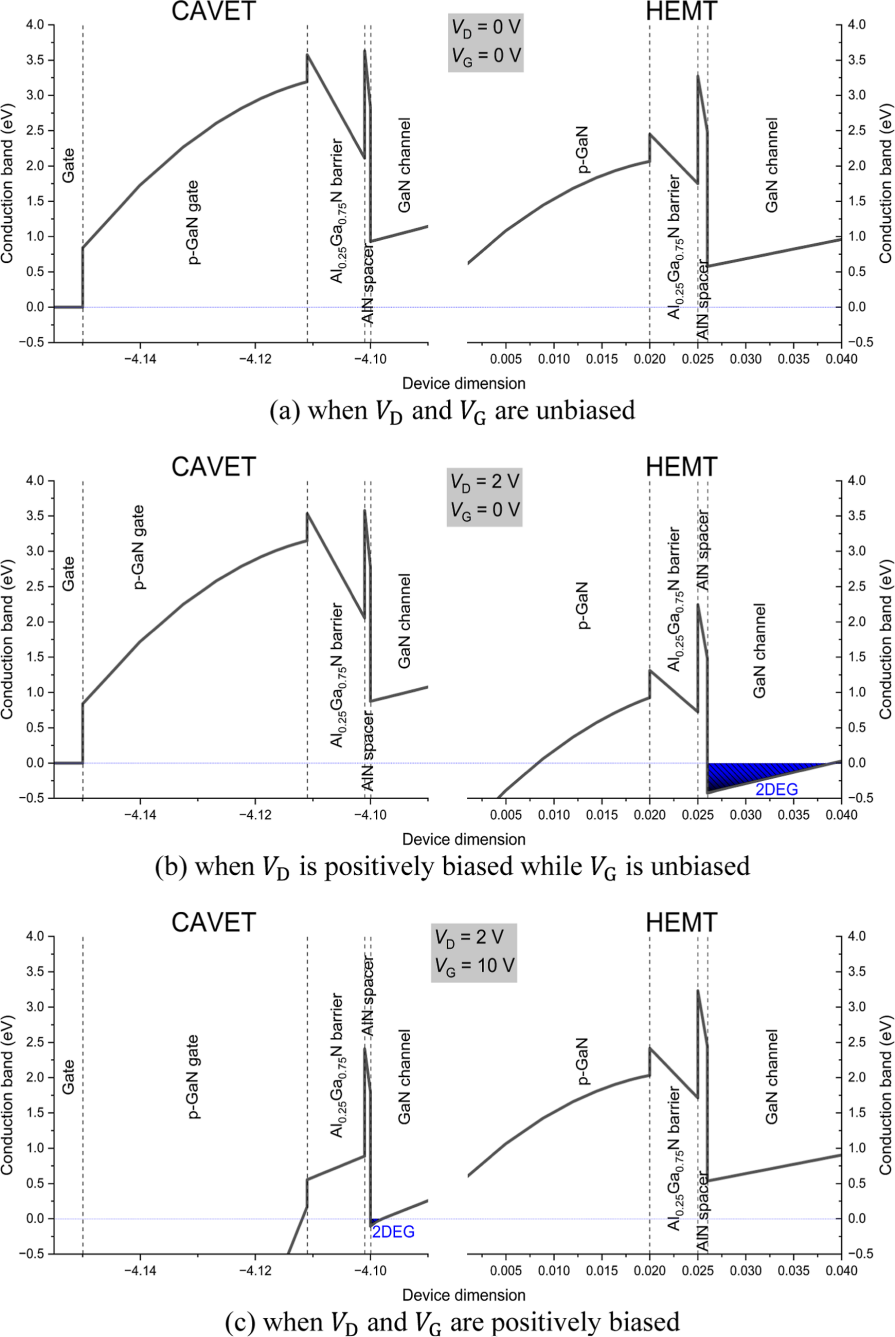
Conduction band energy diagram of the CAVET-HEMT under applied bias.

Figure 4a illustrates the equilibrium condition, where both *V*_D_ and *V*_G_ are unbiased. In this state, the p-GaN layers fully deplete both the CAVET and HEMT regions. The conduction band at the AlN/GaN interfaces remains above the 0 eV reference level, indicating the absence of 2DEG in both lateral and vertical channels. Consequently, no conduction path is established, and the device operates in a normally off state.

As shown in Figure 4b, when a positive drain bias *V*_D_ is applied while the gate remains unbiased, the lateral electric field alters the potential distribution in the HEMT region. The depletion beneath the HEMT p-GaN layer is partially reduced, causing the conduction band to bend below the 0 eV level and enabling 2DEG formation in the lateral channel. The HEMT section therefore turns on. Meanwhile, the CAVET region remains depleted, and the vertical conduction path is still blocked.

Figure 4c depicts the condition when positive drain bias *V*_D_ and positive gate bias *V*_G_ are applied. The depletion beneath the CAVET p-GaN gate is suppressed, shifting the conduction band in the vertical region below the 0 eV level and inducing 2DEG formation along the vertical channel, thereby activating the CAVET section. At the same time, the potential redistribution associated with vertical current conduction restores depletion in the HEMT region, turning the lateral channel off. As a result, the device transitions from lateral conduction to vertical conduction.

The electron concentration profile illustrated in Figure 5 further reinforces the proposed operation mechanism by providing direct physical evidence of the band-controlled transport behavior under applied bias. It exhibits a clear spatial correlation with the conduction band energy distribution, where regions of band lowering correspond to pronounced electron (2DEG) accumulation, while elevated band regions indicate carrier depletion. This clear spatial correspondence demonstrates that bias-induced band modulation governs carrier confinement and channel formation, thereby establishing the vertical-lateral conduction path in the CAVET-HEMT.

**Figure 5:**
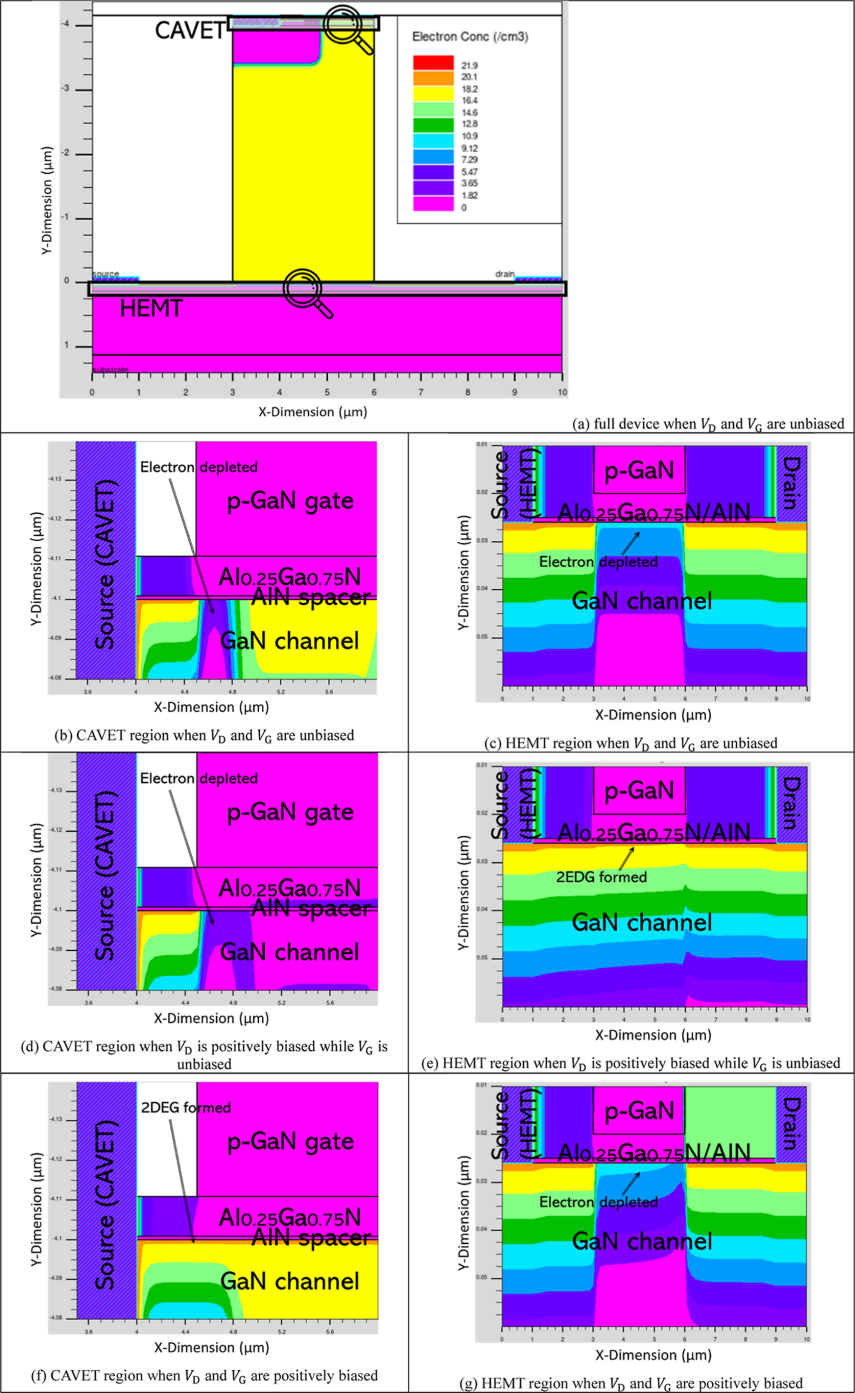
Electron concentration profile of the CAVET-HEMT under applied bias.

## 4 Epitaxial Growth and Fabrication Plan

The process begins with heterostructure growth on a silicon substrate. An AlN nucleation layer is first deposited to mitigate lattice and thermal mismatch between GaN and silicon. An AlGaN buffer layer is subsequently grown to suppress vertical leakage and enhance breakdown capability. An undoped GaN (UID-GaN) channel layer is then formed to provide a high-mobility conduction channel. To further improve carrier confinement and reduce alloy scattering, an AlN spacer layer is inserted above the channel. An AlGaN barrier layer is subsequently grown to induce the two-dimensional electron gas (2DEG) required for lateral HEMT operation. To prepare for the integration of the vertical CAVET section, the AlGaN barrier is selectively etched in the central region, defining the regions for subsequent p-GaN deposition. At this stage, the epitaxial structure of the lateral HEMT is fully established.

Following completion of the lateral HEMT structure, the vertical CAVET section is realized through subsequent epitaxial regrowth. A vertical stack comprising n^+^-GaN and n-GaN layers is regrown to form the charge-balancing and drift regions required for effective vertical electric-field management. The n-GaN drift layer is then selectively etched to enable precise formation of the p-GaN charge-balancing layer (CBL) within the designated region. Subsequently, an unintentionally doped (UID) GaN layer is regrown to achieve the required channel thickness, which is critical for simultaneously sustaining a high breakdown voltage and maintaining low on-state resistance. An AlN layer is regrown above the GaN channel, followed by regrowth of an AlGaN barrier layer to induce the two-dimensional electron gas (2DEG) for CAVET operation. Finally, a p-GaN gate layer is regrown to complete the vertical gate stack. Through these steps, monolithic epitaxial integration of the vertical CAVET onto the lateral HEMT structure is achieved.

In the final phase, device fabrication is carried out through a sequence of selective etching and metallization steps following the predefined device geometry. The AlGaN/AlN layers on both sides of the structure are etched to expose the GaN surface for ohmic contact formation of the lateral HEMT source and drain. Additional selective etching is performed to retain only the necessary CAVET region, with its lateral extent precisely aligned to the p-GaN layer of the HEMT. Within the CAVET section, the AlGaN barrier and n-GaN channel layers are selectively etched to open contact windows for CAVET source metallization. The p-GaN gate layer on top of the CAVET is then selectively etched to define the gate region according to the final device layout. Finally, metal deposition is performed to form the source, gate, and drain electrodes, resulting in the fully functional monolithic CAVET-HEMT structure shown in Figure 6.

**Figure 6:**
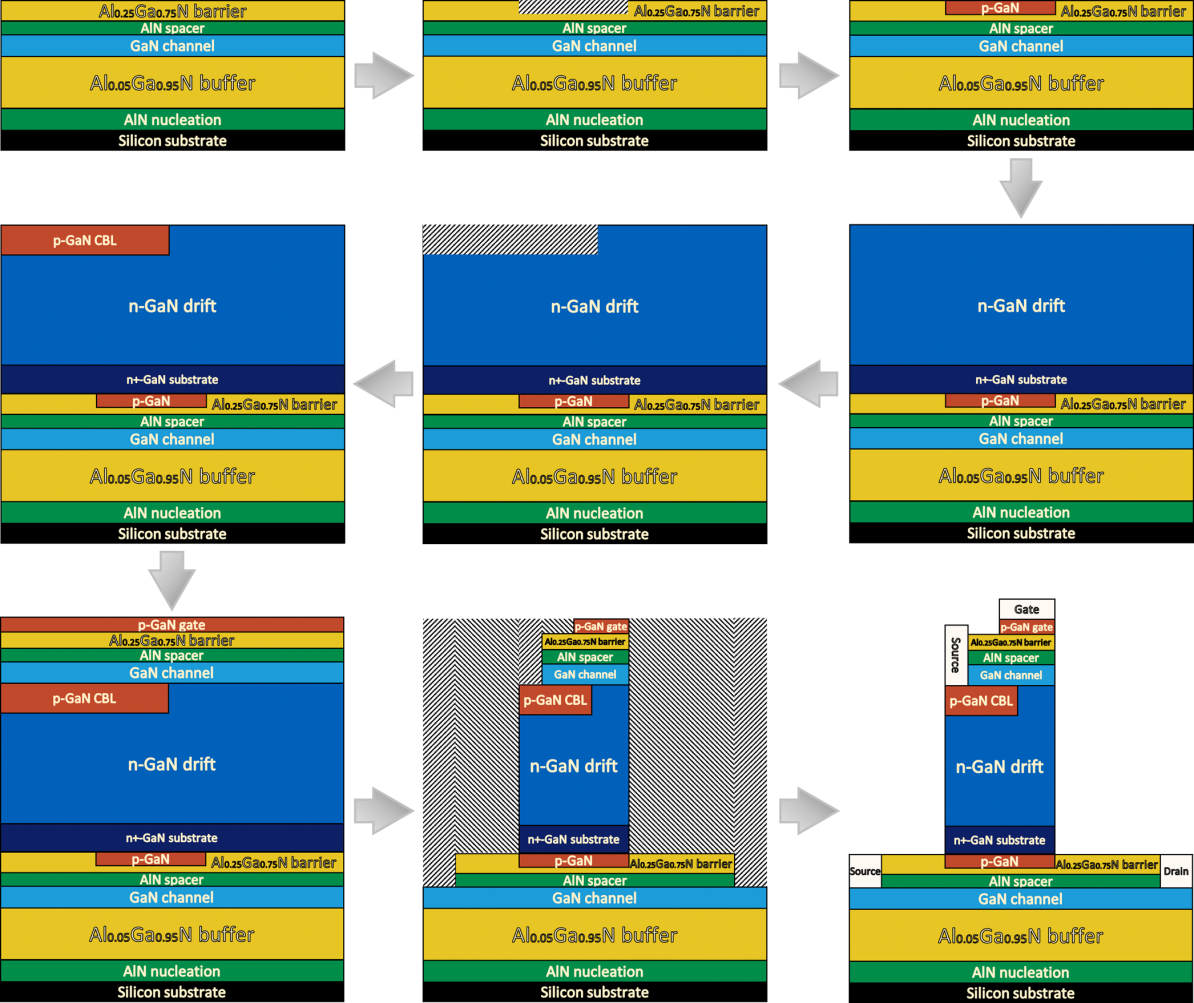
Epitaxial growth and fabrication process flow for the CAVET-HEMT.

While the outlined design is compatible with current epitaxial regrowth and microfabrication processes, the multistep fabrication introduces several practical challenges. Slight variations in the thickness of the UID-GaN channel or p-GaN charge-balancing layer may degrade carrier confinement, potentially increase on-state resistance or reduce breakdown voltage. Defects at regrowth interfaces can introduce scattering centers or traps, which may shift the threshold voltage and reduce 2DEG mobility. Precise alignment between the lateral HEMT and vertical CAVET regions is critical, as misalignment can produce nonuniform electric fields, increasing leakage current and locally lowering breakdown performance. In addition, over- or under-etching during selective patterning of barrier and channel layers may affect gate control, leading to threshold voltage variability and reduced device uniformity. Overall speaking, these practical process deviations could result in measurable performance variations in breakdown voltage, threshold voltage, and on-state resistance. Nevertheless, with advanced epitaxial regrowth techniques, high-resolution lithography, and precise etching control, the proposed monolithic CAVET-HEMT structure remains fabrication-feasible, and the expected performance deviations can be mitigated to maintain device functionality.

## 5 Results and Discussion

Figure 7a shows the *I*_D_–*V*_G_ curves of the CAVET-HEMT compared with a HEMT at different drain voltages *V*_D_. The HEMT has a threshold voltage of *V*_th_ = 0.9 V, which marks the gate voltage above which the device starts to conduct. In contrast, the CAVET-HEMT has a threshold voltage of *V*_th_ = 1.1 V, but its behavior is inverted, where the device conducts at gate voltages below the threshold and is fully off when *V*_G_ exceeds this threshold. This highlights the novel inverting-switch functionality of the CAVET-HEMT.

**Figure 7:**
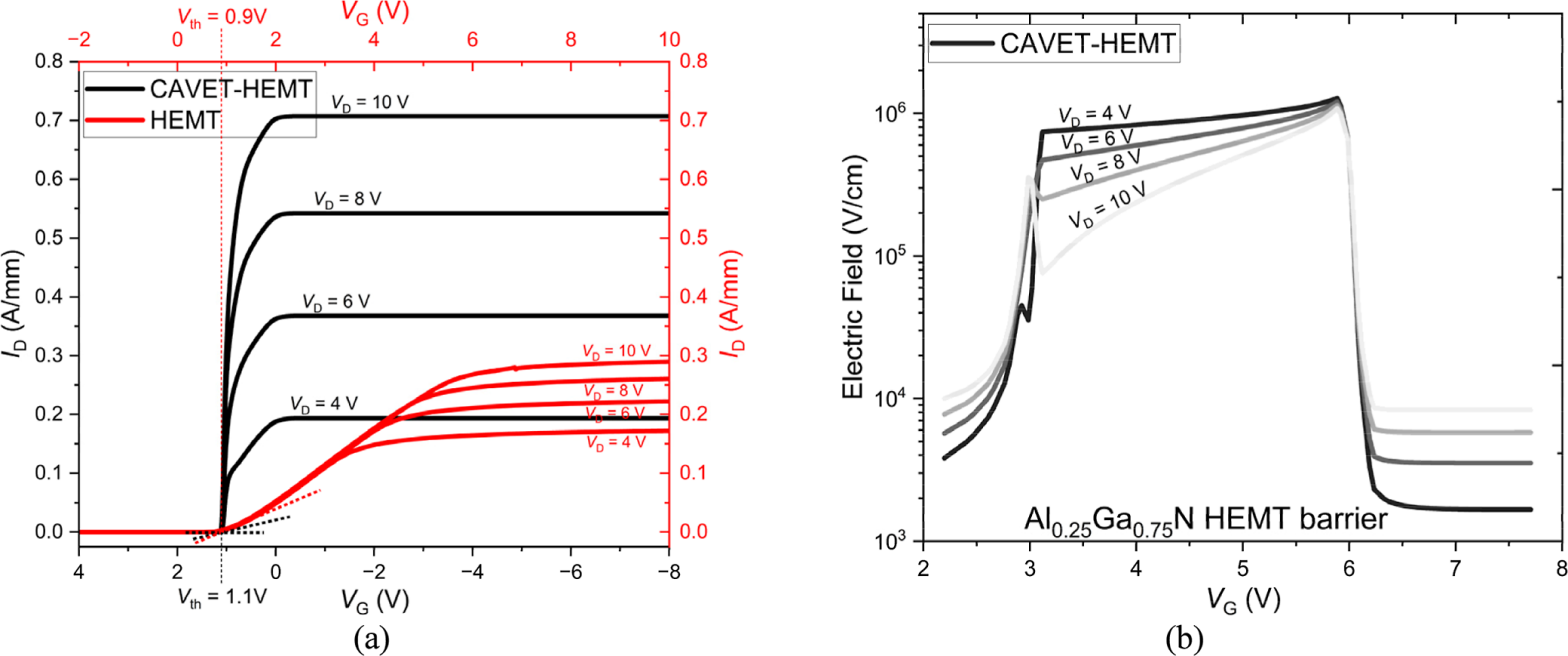
(a) *I*_D_–*V*_G_ characteristic. (b) Electric field–*V*_G_ characteristic.

In addition, the CAVET-HEMT exhibits a consistently higher saturation drain current *I*_D,sat_ than the HEMT across all tested drain voltages *V*_D_. More importantly, the scaling of *I*_D,sat_ with increasing *V*_D_ is substantially stronger in the CAVET-HEMT. At *V*_D_ = 4 V, the CAVET-HEMT delivers *I*_D,sat_ of 0.193 A/mm compared to 0.172 A/mm for the HEMT. As *V*_D_ increases to 6, 8, and 10 V, the *I*_D,sat_ of the CAVET-HEMT rises to 0.368 A/mm, 0.542 A/mm, and 0.707 A/mm, respectively, whereas the HEMT reaches only 0.222 A/mm, 0.260 A/mm, and 0.290 A/mm. This progressively widening current gap at higher drain bias reflects the enhanced current-driving capability.

Following the *I*_D_–*V*_G_ characteristics, Figure 8a,b present the electric field distribution, providing insight into the device operation. The spatial profiles reveal a pronounced electric field peak at the edge of the p-GaN region near the drain. This peak results from electric field crowding at the sharp gate-edge geometry: the small radius of curvature causes field lines to converge, producing enhanced local surface charge density and a stronger electric field.

**Figure 8:**
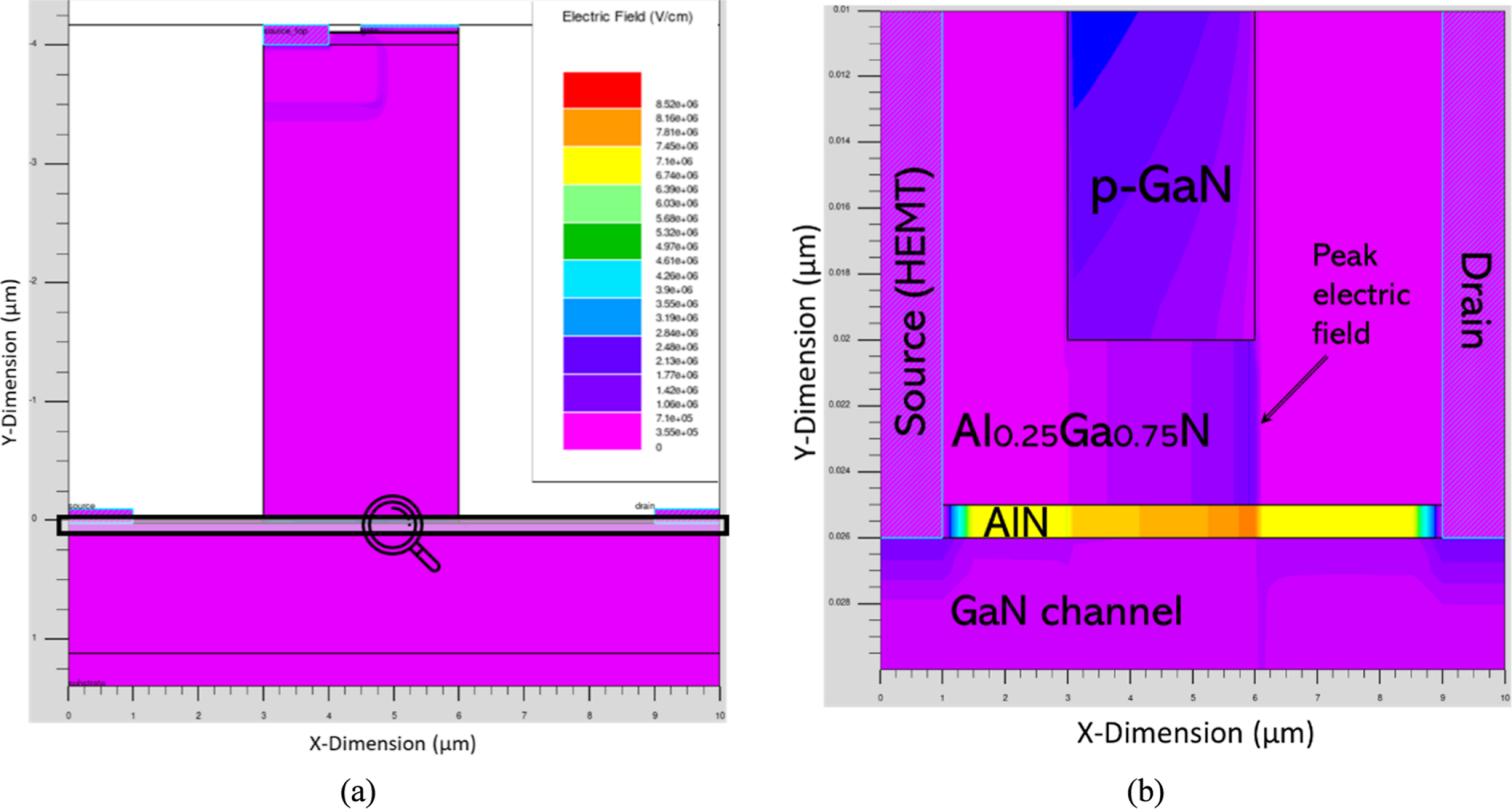
(a) Electric field distribution profile of CAVET-HEMT at positive biased *V*_D_. (b) Magnified view of highlighted region in (a).

To investigate how this localized field evolves under operating conditions, Figure 7b presents the electric field distribution within the Al_0.25_Ga_0.75_N HEMT barrier region at increasing drain voltages. As the drain bias rises, the high-field region gradually spreads toward the drain, redistributing the field along the channel. This spatial spreading reduces the localization of the electric field at the p-GaN (gate) edge, lowering the peak field. Simultaneously, the field profile becomes less uniform due to enhanced lateral field penetration and drain-side channel pinch-off at higher bias. This enables more effective carrier acceleration before velocity saturation occurs, thereby increasing the saturation drain current *I*_D,sat_.

Notably, the *I*_D_–*V*_G_ curves of the CAVET-HEMT exhibit a much steeper slope than that of the HEMT, whose transfer characteristic appears comparatively gradual. Since transconductance (*g*_m_) is defined as the derivative ∂*I*_D_/∂*V*_G_, a steeper slope directly corresponds to higher transconductance, indicating more efficient conversion of gate-voltage variation into drain-current response. Figure 9a presents the extracted *g*_m_ characteristics for both devices. The HEMT exhibits a conventional bell-shaped *g*_m_ curve with relatively low and nearly constant peak values. In contrast, the CAVET-HEMT shows a sharply peaked *g*_m_ profile confined to a narrow gate-voltage range. The narrow and sharp *g*_m_ characteristic of the CAVET-HEMT indicates a rapid transition between conduction states, which is advantageous for high-speed switching and high-gain operation due to stronger gate sensitivity. Quantitatively, the CAVET-HEMT achieves peak |*g*_m_| values of 1.117 S/mm, 2.283 S/mm, 3.010 S/mm, and 4.296 S/mm at *V*_D_ = 4, 6, 8, and 10 V, respectively. The corresponding values for the HEMT remain nearly unchanged at approximately 0.062–0.064 S/mm over the same drain-voltage range. This substantial difference confirms that the CAVET-HEMT provides significantly stronger gate control and enhanced current modulation capability.

**Figure 9:**
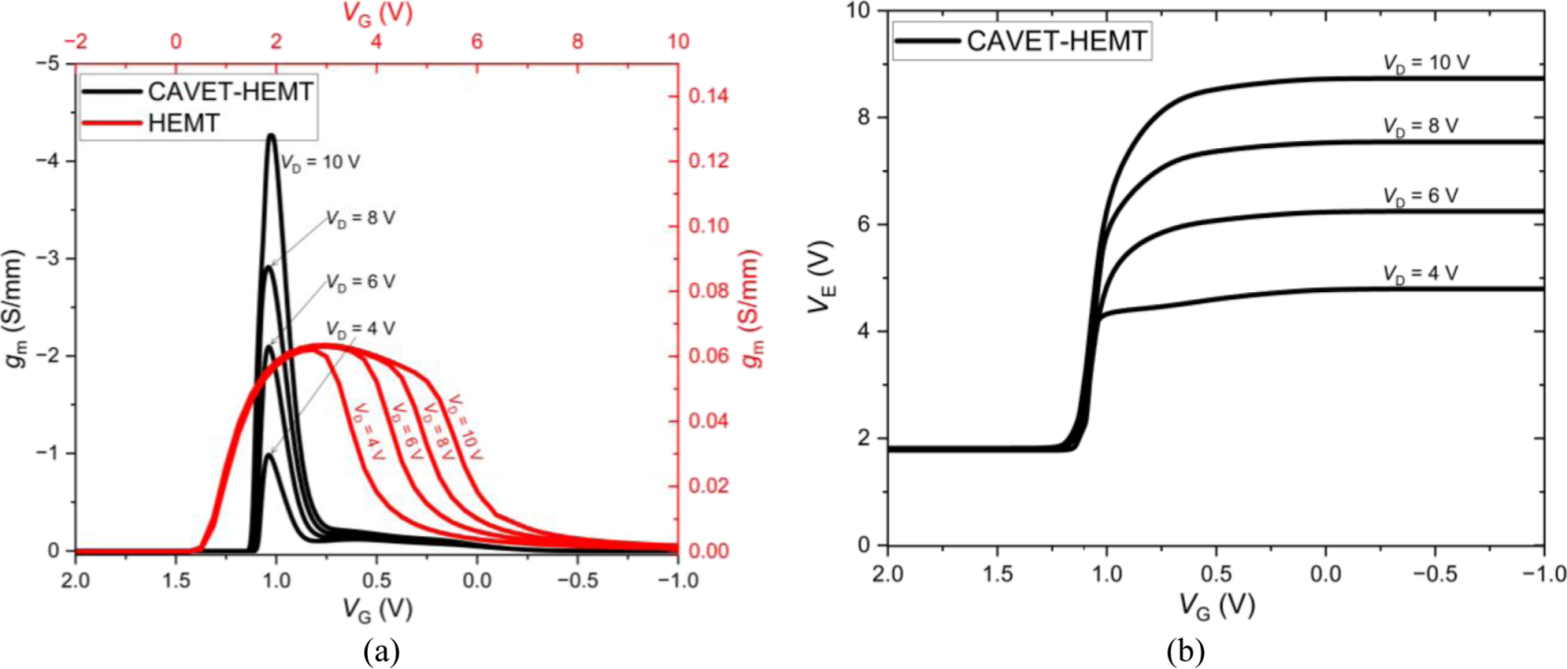
(a) *g*_m_–*V*_G_ characteristic. (b) *V*_E_–*V*_G_ characteristic.

Accordingly, the voltage gain (*A*_v_) contributed by the CAVET region can be approximated from the ratio of the two triode (active) voltage ranges as follows

1 $$A_{v}=\frac{\Delta V_{triode\left(HEMT\right)}}{\Delta V_{triode\left(CAVET‐HEMT\right)}}$$

where Δ*V*_triode_ is defined as the gate-voltage interval over which *g*_m_ remains significant and useable. At *V*_D_ = 10 V, the CAVET-HEMT exhibits an active range from 1.25 to 0.1 V, corresponding to Δ*V*_triode(CAVET‑HEMT)_ = 1.15 V. In contrast, the HEMT shows an active range from 0.75 to 7 V, yielding Δ*V*_triode(HEMT)_ = 6.25 V. Using the extracted values, eq 1 yields an estimated voltage gain of approximately *A*_v_ = 5.43. This indicates that the CAVET region effectively enhances the gate-voltage control sensitivity by a factor of 5.43 within the integrated architecture. In practical terms, a 1 V variation at the CAVET input corresponds to an equivalent gate-control modulation of approximately 5.43 V at the internal HEMT region, thereby confirming the intrinsic voltage amplification capability of the CAVET-HEMT structure.

Here, we relate the potential (*V*_E_) distribution inside the CAVET-HEMT to its operation. Figure 10a presents the electrostatic potential profile at *V*_D_ = 10 V and *V*_G_ = 1.0 V, while Figure 10b provides a magnified view of the highlighted region. A pronounced potential drop is observed near the edge of the p-GaN layer, caused by strong electric field concentration at the gate termination (see Figure 8b). This localized high field induces enhanced depletion and modulates the vertical-lateral potential distribution, leading to the formation of a high-resistance region at the p-GaN/Al_0.25_Ga_0.75_N interface. This high-resistance region is critical for the device’s intrinsic inverting-switch operation, enabling both 180° phase inversion and voltage amplification when the CAVET component is cascaded with or integrated onto the HEMT.

**Figure 10:**
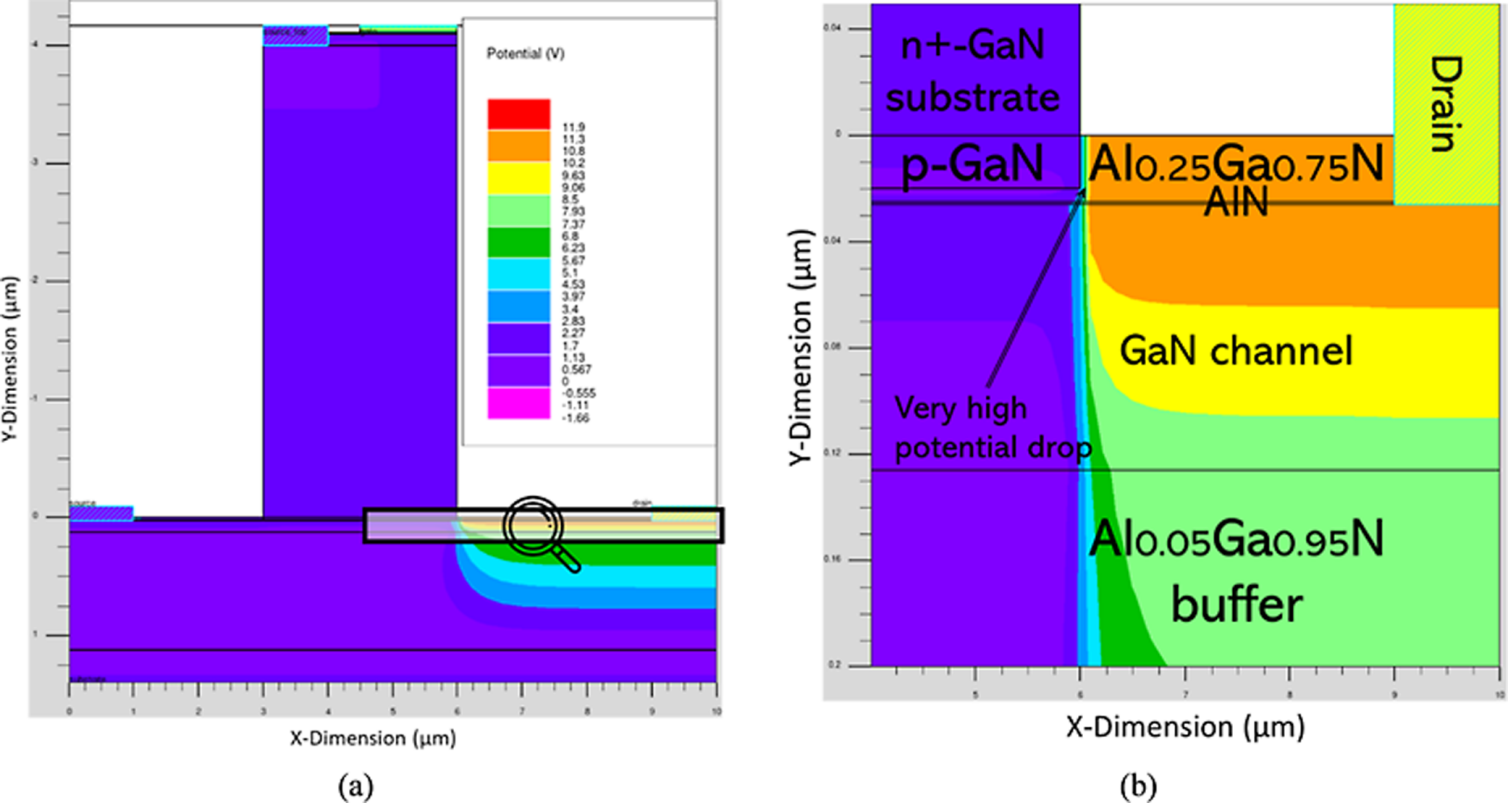
(a) Potential *V*_E_ distribution profile of CAVET-HEMT at *V*_D_ = 10 V and *V*_G_ = 1.0 V. (b) Magnified view of highlighted region in (a).

The *V*_E_–*V*_G_ characteristics at the p-GaN edge (see Figure 9b), measured under increasing drain voltage, provide further insight into the device behavior. The peak potential at the p-GaN edge is measured to be 4.797, 6.247, 7.547, and 8.733 V for *V*_D_ = 4, 6, 8, and 10 V, respectively. As the drain voltage increases, the absolute potential at the p-GaN edge rises, steepening the local potential gradient along the vertical-lateral path. This behavior explains why the transconductance becomes sharper and reaches higher values, as the potential at the p-GaN edge reflects the gate’s control over the lateral channel. A steeper potential gradient improves gate control sensitivity and leads to higher transconductance. Interestingly, this increase in potential occurs alongside a reduction in the peak electric field (see Figure 7b) at higher drain biases. This inverse behavior occurs because the high-field region spreads toward the drain, reducing the local peak field and allowing the channel near the drain to support a higher potential.

Figure 11 presents the *I*_D_–*V*_D_ characteristics of the CAVET-HEMT compared with the HEMT at different gate voltages *V*_G_. A clear distinction is observed in the onset behavior of the output curves. For the HEMT, all *I*_D_–*V*_D_ curves originate from *V*_D_ = 0 V, indicating that current conduction is solely governed by the applied gate voltage. In contrast, the CAVET-HEMT exhibits a gate-dependent shift in the apparent turn-on point along the *V*_D_ axis, where higher *V*_G_ results in a larger initial *V*_D_ required for current rise. This behavior reflects the cascaded architecture of the CAVET-HEMT. Unlike a HEMT, where conduction is directly controlled by *V*_G_, the CAVET-HEMT involves two coupled mechanisms: the drain voltage partially activates the lower HEMT section, while the gate voltage controls the upper CAVET section. As a result, the drain bias itself contributes to device initiation, leading to the distinct output characteristics observed. The device operation therefore depends on the combined influence of both *V*_D_ and *V*_G_, rather than purely gate control.

**Figure 11:**
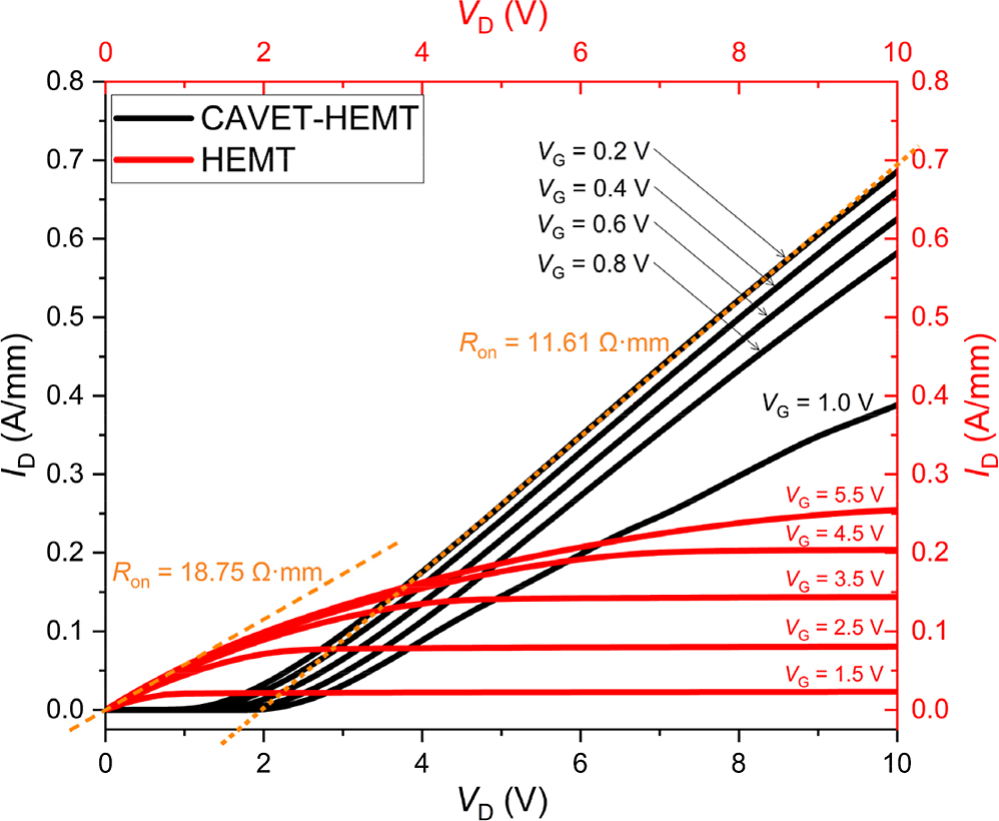
*I*_D_–*V*_D_ characteristic.

Another important observation is the delayed saturation behavior of the CAVET-HEMT. The output current continues to increase with *V*_D_ and does not rapidly converge to saturation. In contrast, the HEMT transitions from the triode region to saturation at comparatively lower drain voltages. This extended current rise in the CAVET-HEMT enables significantly higher *I*_D,max_, consistent with the trends previously discussed.

The on-resistance *R*_on_ is extracted from the inverse slope of the linear region of the *I*_D_–*V*_D_ curve, typically evaluated under a fully on gate bias to ensure minimal channel resistance contribution from incomplete conduction. From the extracted data, the CAVET-HEMT exhibits *R*_on_ = 11.61 Ω·mm, whereas the HEMT shows *R*_on_ = 18.75 Ω·mm. The lower *R*_on_in the CAVET-HEMT indicates reduced channel resistance and improved current conduction efficiency.

The output conductance *g*_d_ is defined as the derivative ∂*I*_D_/∂*V*_D_, evaluated in the triode region. Figure 12 presents the extracted *g*_d_ as a function of *V*_D_. A higher *g*_d_ indicates that the drain current *I*_D_ remains more sensitive to variations in the drain voltage *V*_D_ within the triode region. It should be noted that these gate-bias values are selected from the beginning to near the end of the triode region for each device. Therefore, the comparison reflects the output conductance behavior across the effective linear operating range rather than at arbitrary bias points. For the CAVET-HEMT, the peak *g*_d_ values are 0.088 S/mm, 0.087 S/mm, 0.085 S/mm, 0.082 S/mm, and 0.061 S/mm at *V*_G_ = 0.2, 0.4, 0.6, 0.8, and 1.0 V, respectively. In comparison, the HEMT exhibits peak *g*_d_ values of 0.0012 S/mm, 0.0465 S/mm, 0.0527 S/mm, 0.0557 S/mm, and 0.0575 S/mm at *V*_G_ = 1.5, 2.5, 3.5, 4.5, and 5.5 V, respectively. Overall, the CAVET-HEMT consistently shows higher *g*_d_ than the HEMT. In addition, the evolution trend differs between the two devices: the CAVET-HEMT exhibits an initially increasing *g*_d_ behavior with *V*_D_, whereas the HEMT shows a decreasing trend from the onset of saturation. This distinction further highlights the different output characteristics arising from their structural and operational mechanisms.

**Figure 12:**
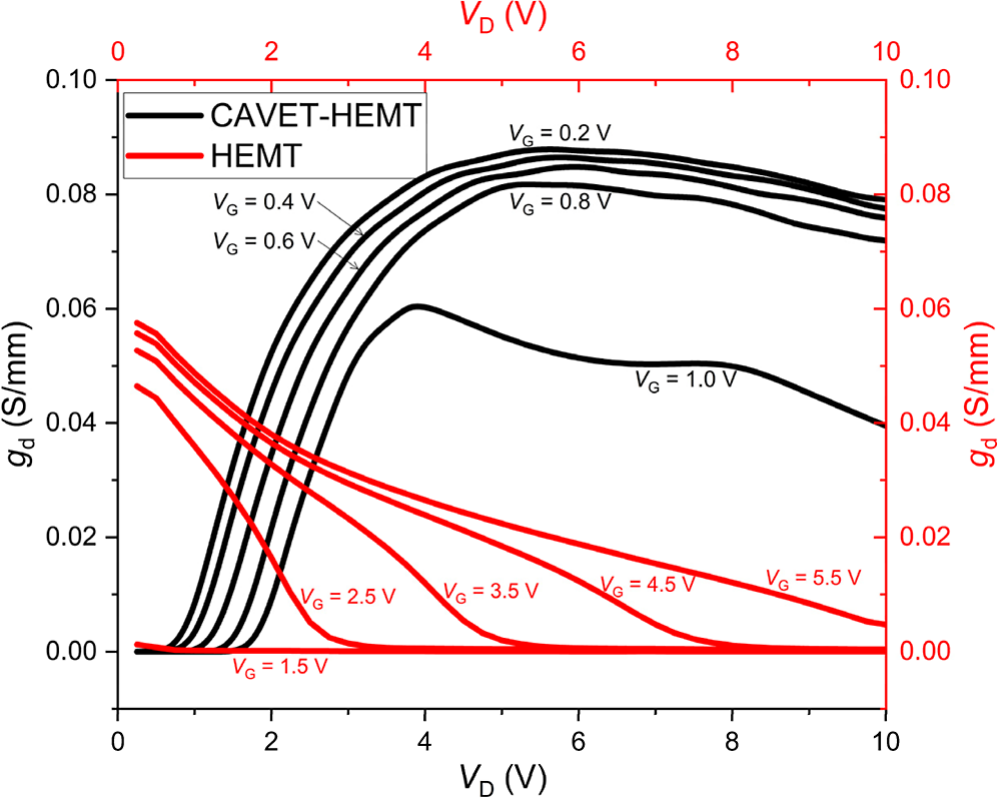
*g*_d_–*V*_D_ characteristic.

According to the intrinsic gain expression

2 $$A_{0}=\frac{\left|g_{m}\right|}{g_{d}}$$

an increase in *g*_d_ directly reduces the intrinsic gain *A*_0_. Consequently, the relatively higher output conductance of the CAVET-HEMT leads to a lower intrinsic gain compared to the conventional HEMT. This statement is confirmed in Figure 13, which plots the intrinsic gain versus gate voltage *V*_G_ for both devices.

**Figure 13:**
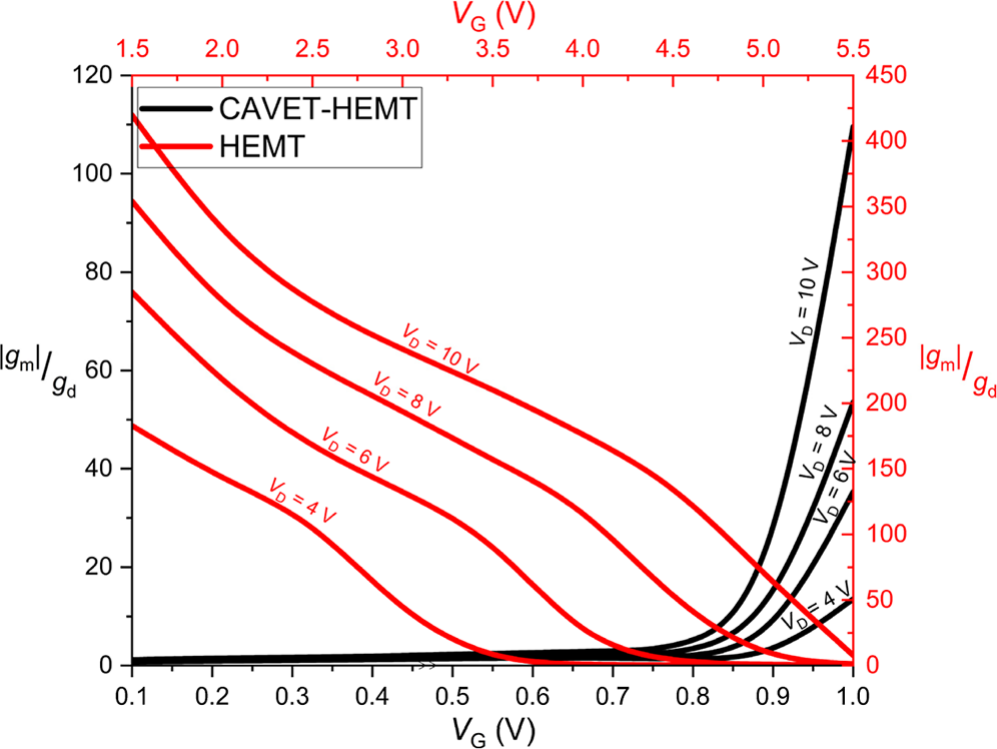
*A*_0_–*V*_G_ characteristic.

More specifically, the intrinsic gain *A*_0_of the CAVET-HEMT increases with gate voltage *V*_G_, highlighting its inverted operating mode and confirming its function as an inverting switch rather than a normal HEMT. Quantitatively, at *V*_D_ = 4 V, the CAVET-HEMT reaches a peak intrinsic gain of 13.56, compared to 182.99 for the HEMT. At *V*_D_ = 6 V, the peak gains are 35.35 and 284.73; at *V*_D_ = 8 V, 53.57 and 354.14; and at *V*_D_ = 10 V, 109.59 and 419.84, respectively. Overall, the absolute intrinsic gain of the CAVET-HEMT is lower than that of the HEMT. Since intrinsic gain represents the maximum achievable voltage gain of a single isolated device, the lower *A*_0_ of the CAVET-HEMT indicates a trade-off between its enhanced current-driving capability and its small-signal amplification performance.

According to standard relationships, the current-gain cutoff frequency *f*_T_ and the maximum oscillation frequency *f*_max_ are given by

3 $$f_{T}=\frac{g_{m}}{2\pi \left(C_{GS}+C_{GD}\right)}$$

4 $$f_{\max}=\frac{f_{T}}{2\sqrt{\frac{R_{G}}{g_{m}}\left(C_{GS}+C_{GD}\right)+R_{S}C_{GS}+R_{D}C_{GD}}}$$

where *C*_GS_ and *C*_GD_ are the gate-source and gate-drain capacitances, and *R*_G_, *R*_S_, and *R*_D_ are the gate, source, and drain resistances, respectively. These expressions highlight that both *f*_T_ and *f*_max_ are strongly influenced by the transconductance *g*_m_ and the parasitic capacitances. Conceptually, integrating the CAVET region increases the total parasitic capacitance of the device in addition to the inherent *C*_GS_and *C*_GD_, which could potentially degrade high-frequency performance. Nevertheless, the CAVET region’s voltage amplification enhances transconductance (*g*_m_), providing a compensatory improvement in device response.

Figure 14a,b present the frequency-dependent characteristics of the CAVET-HEMT and HEMT devices. Figure 14a shows the current gain versus frequency, from which *f*_T_ is determined as the frequency at which the current gain drops to unity (0 dB). Figure 14b shows the power gain versus frequency, from which *f*_max_ is extracted, corresponding to the frequency at which the power gain falls to unity (0 dB). For the CAVET-HEMT, *f*_T_ = 3.5 GHz and *f*_max_ = 6.5 GHz, while the HEMT exhibits *f*_T_ = 3.1 GHz and *f*_max_ = 6.0 GHz. The slight improvement in both *f*_T_ and *f*_max_ for the CAVET-HEMT indicates that the enhanced *g*_m_ effectively compensates for the additional parasitic capacitance, producing a net positive impact on high-frequency performance. In other words, the device leverages the CAVET-induced transconductance enhancement to overcome the parasitic burden, demonstrating that the integrated architecture can improve speed and gain without being significantly limited by added capacitances.

**Figure 14:**
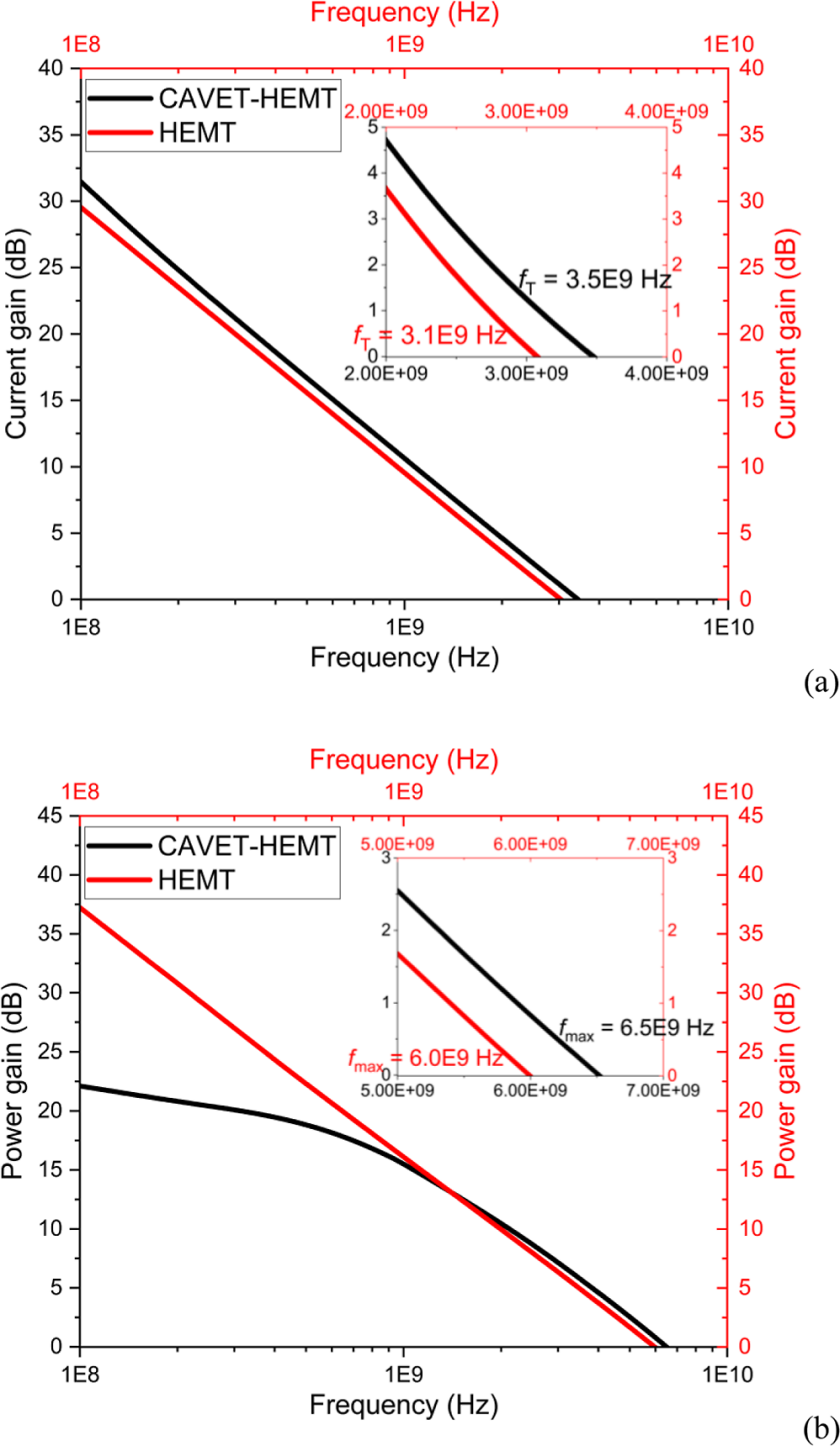
RF output characteristics: (a) current gain over frequency. (b) Power gain over frequency.

In summary, Table 2 provides a consolidated overview of the key electrical characteristics previously discussed, allowing a clear comparison between the proposed CAVET-HEMT and the HEMT.

**Table 2:** Summary of DC and RF Performance of the CAVET-HEMT

	HEMT	proposed CAVET-HEMT
function	normal switch	inverting switch
*V*_th_	0.9 V	1.1 V
*I*_D,sat_	0.290 A/mm	0.707 A/mm
peak |*g*_m_|	0.064 S/mm	4.296 S/mm
Δ*V*_triode_	6.25 V	1.15 V
*R*_on_	18.75 Ω·mm	11.61 Ω·mm
peak *g*_d_	0.0575	0.088 S/mm
peak intrinsic gain	354.14	53.57
*f*_T_	3.1 GHz	3.5 GHz
*f*_max_	6.0 GHz	6.5 GHz
parasitic capacitance	lower	higher

## 6 Conclusion and Future Prospects

This work introduces a monolithic GaN-based CAVET-HEMT, a completely novel device architecture that integrates a vertical CAVET directly onto a lateral HEMT. By embedding cascading behavior within the device structure and incorporating a 180° phase inversion, the CAVET-HEMT operates as an inverting switch, fundamentally different from conventional HEMTs. This internal cascade enables the CAVET region to modulate the potential delivered to the HEMT, compressing the operating voltage range and amplifying gate-control sensitivity, without the need for external circuits. Electrical characterization confirms the device’s advantages. The CAVET-HEMT has a threshold voltage *V*_th_ of 1.1 V, above which the device fully turns off. Its saturation current *I*_D,sat_ reaches 0.707 A/mm, more than double that of the HEMT, reflecting strong current-driving capability. The peak transconductance |*g*_m_| of 4.296 S/mm, combined with a narrow triode operating range Δ*V*_triode_ of 1.15 V, demonstrates enhanced voltage amplification and high sensitivity, compared to the broader 6.25 V range in the HEMT. In addition, the CAVET-HEMT exhibits low on-resistance (*R*_on_ = 11.61 Ω·mm) and higher output conductance (*g*_d_ = 0.088 S/mm), supporting efficient conduction while maintaining controllability. Despite the increased parasitic capacitance inherent to the integrated CAVET structure, the device achieves slightly improved high-frequency performance with *f*_T_ = 3.5 GHz and *f*_max_ = 6.5 GHz, showing that the transconductance enhancement effectively compensates for parasitic effects. These results confirm that the monolithic integration enhances current-handling, gain modulation, and frequency response simultaneously. Beyond numerical performance, the CAVET-HEMT introduces a new operational paradigm. Its inverting-switch functionality enables compact, high-speed, and high-power switching while inherently embedding logic capability, potentially reducing circuit complexity and parasitic losses. By consolidating multistage switching behavior within a single device, the CAVET-HEMT opens new possibilities for advanced GaN-based electronics.

Looking ahead, the next research phase focuses on device fabrication and regrowth processes to realize the CAVET-HEMT experimentally and validate its performance. Particular focus will be placed on testing its applicability as a NOT logic gate, including inverting switching behavior, reliability, and integration potential within functional circuits. Further optimization of structural parameters, thermal management, and scalable fabrication techniques will be essential to maximize the device’s practical applicability and performance in real-world applications.

## References

[1] Alim M. A., Chowdhury A. Z., Islam S., Gaquiere C., Crupi G. (2021). Temperature-Sensitivity
of Two Microwave HEMT Devices: AlGaAs/GaAs vs. AlGaN/GaN Heterostructures. Electronics.

[2] Banerjee, A. AlGaAs-GaAs, AlGaN-GaN, SiC, HEMT Large Signal Equivalent Electrical Circuits (Angelov Chalmers Model)-Normally On|Off HEMT pHEMT, mHEMT and MODFET. Semiconductor Devices. Synthesis Lectures on Engineering, Science, and Technology; Springer, 2024; pp 91–102.

[3] Lenka T. R., Panda A. K. (2011). Characteristics
study of 2DEG transport properties
of AlGaN/GaN and AlGaAs/GaAs-based HEMT. Semiconductors.

[4] Dutta, R. ; Tamang, T. ; Paul, P. ; Paitya, N. Comparative Study of AlGaN/GaN, InAlN/GaN and AlGaAs/GaAs based High Electron Mobilty Transistors Using Silvaco for High-Frequency Applications. 2020 4th International Conference on Trends in Electronics and Informatics (ICOEI)(48184); IEEE, 2020; pp 137–142.

[5] Zeng F., An J. X., Zhou G., Li W., Wang H., Duan T., Jiang L., Yu H. (2018). A Comprehensive
Review
of Recent Progress on GaN High Electron Mobility Transistors: Devices,
Fabrication and Reliability. Electronics.

[6] Liu C.-H., Chiu H.-C., Wang H.-C., Kao H.-L., Huang C.-R. (2021). Improved
Gate Reliability Normally-Off p-GaN/AlN/AlGaN/GaN HEMT With AlGaN
Cap-Layer. IEEE Electron Device Lett..

[7] Kalita S., Awadhiya B., Changmai P. (2023). Performance
analysis of gallium nitride-based
DH-HEMT with polarization-graded AlGaN back-barrier layer. Appl. Phys. B Laser Opt..

[8] Yang J., Wei J., Wu Y., Nuo M., Chen Z., Yang X., Wang M., Shen B. (2023). 600-V p-GaN
Gate HEMT With Buried
Hole Spreading Channel Demonstrating Immunity Against Buffer Trapping
Effects. IEEE Electron Device Lett..

[9] Fletcher A. S. A., Nirmal D. (2017). A survey of Gallium
Nitride HEMT for RF and high power
applications. Superlattices Microstruct..

[10] Mounika B., Ajayan J., Bhattacharya S. (2023). An intensive
study on effects of
lateral scaling and gate metals on the RF/DC performance of recessed
T-gated Fe-doped AlN/GaN/SiC HEMTs for future RF and microwave power
applications. Microelectron. Eng..

[11] Hu J., Zhang Y., Sun M., Piedra D., Chowdhury N., Palacios T. (2018). Materials and processing issues in vertical GaN power
electronics. Mater. Sci. Semicond. Process..

[12] Ji D., Agarwal A., Li W., Keller S., Chowdhury S. (2018). Demonstration
of GaN Current Aperture Vertical Electron Transistors With Aperture
Region Formed by Ion Implantation. IEEE Trans.
Electron Devices.

[13] Wen X., Lee K. J., Nakazato Y., Chun J., Chowdhury S. (2023). High Current
Density Trench CAVET on Bulk GaN Substrates with Low-Temperature GaN
Suppressing Mg Diffusion. Crystals.

[14] Li Y., Xu L., Guo Z., Sun H. (2022). Study of High-Performance GaN-Based
Trench CAVET with Stepped Doping Microstructure. Micromachines.

[15] Zhang M. (2021). Study of AlGaN/GaN Vertical
Superjunction HEMT for Improvement of
Breakdown Voltage and Specific On-Resistance. IEEE Access.

[16] M, J. N. ; M, T. ; G, A. F. ; Ramaraj, K. Improvement of the Effective Bandwidth of Multistage Amplifier by Cascading the Individual Two Stage Feedback Amplifiers. 2023 International Conference on Self Sustainable Artificial Intelligence Systems (ICSSAS); IEEE, 2023; pp 1714–1718.

[17] Poole, C. ; Darwazeh, I. Microwave amplifier design. Microwave Active Circuit Analysis and Design; Elsevier, 2016; pp 439–473.

[18] Tarar M. M., Qayyum S., Ali A., Negra R. (2024). Loss-Compensated
Cascaded
Multistage Distributed Power Amplifier in 65nm CMOS Technology. IEEE Access.

[19] Trinh V.-S., Song J.-M., Park J.-D. (2022). A 280 GHz
30 GHz Bandwidth Cascaded
Amplifier Using Flexible Interstage Matching Strategy in 130 nm SiGe
Technology. Electronics.

[20] Lin R., Zhao D., Yu G., Liu X., Wu D., Gu E., Cui X., Liu R., Zhang B., Tian P. (2020). Fabrication
and characteristics of flexible normally-off AlGaN/GaN HEMTs. AIP Adv..

[21] ATLAS User’s Manual 2023; Silvaco Inc: 4701 Patrick Henry Drive, Building 1 Santa Clara, CA 95054, USA, 2023.

